# Characterization of Phosphofructokinase Activity in *Mycobacterium tuberculosis* Reveals That a Functional Glycolytic Carbon Flow Is Necessary to Limit the Accumulation of Toxic Metabolic Intermediates under Hypoxia

**DOI:** 10.1371/journal.pone.0056037

**Published:** 2013-02-07

**Authors:** Wai Yee Phong, Wenwei Lin, Srinivasa P. S. Rao, Thomas Dick, Sylvie Alonso, Kevin Pethe

**Affiliations:** 1 Novartis Institute for Tropical Diseases, Singapore, Singapore; 2 Department of Microbiology, Immunology Programme, Yong Loo Lin School of Medicine, Life Sciences Institute, National University of Singapore, Singapore, Singapore; 3 Singapore-Massachusetts Institute of Technology Alliance for Research and Technology (SMART), CREATE NUS Campus, Singapore, Singapore; Infectious Disease Research Institute, United States of America

## Abstract

Metabolic versatility has been increasingly recognized as a major virulence mechanism that enables *Mycobacterium tuberculosis* to persist in many microenvironments encountered in its host. Glucose is one of the most abundant carbon sources that is exploited by many pathogenic bacteria in the human host. *M. tuberculosis* has an intact glycolytic pathway that is highly conserved in all clinical isolates sequenced to date suggesting that glucose may represent a non-negligible source of carbon and energy for this pathogen *in vivo*. Fructose-6-phosphate phosphorylation represents the key-committing step in glycolysis and is catalyzed by a phosphofructokinase (PFK) activity. Two genes, *pfkA* and *pfkB* have been annotated to encode putative PFK in *M. tuberculosis*. Here, we show that PFKA is the sole PFK enzyme in *M. tuberculosis* with no functional redundancy with PFKB. PFKA is required for growth on glucose as sole carbon source. In co-metabolism experiments, we report that disruption of the glycolytic pathway at the PFK step results in intracellular accumulation of sugar-phosphates that correlated with significant impairment of the cell viability. Concomitantly, we found that the presence of glucose is highly toxic for the long-term survival of hypoxic non-replicating mycobacteria, suggesting that accumulation of glucose-derived toxic metabolites does occur in the absence of sustained aerobic respiration. The culture medium traditionally used to study the physiology of hypoxic mycobacteria is supplemented with glucose. In this medium, *M. tuberculosis* can survive for only 7–10 days in a true non-replicating state before death is observed. By omitting glucose in the medium this period could be extended for up to at least 40 days without significant viability loss. Therefore, our study suggests that glycolysis leads to accumulation of glucose-derived toxic metabolites that limits long-term survival of hypoxic mycobacteria. Such toxic effect is exacerbated when the glycolytic pathway is disrupted at the PKF step.

## Introduction

Despite the availability of effective anti-tubercular drugs, tuberculosis (TB) remains a scourge of public health with 8.8 million people infected with active TB in 2010 [1]. The metabolic versatility of *M. tuberculosis*, the etiological agent of TB, represents one of the key virulence strategies developed by the pathogen to persist in many microenvironments within its host [2]. Earlier studies on carbon metabolism have shown that *M. tuberculosis* can utilize a variety of carbon substrates [3].

During infection, several studies have shown that the gluconeogenic pathway is required for infection and persistence, suggesting that fatty acids constitute one of the main carbon and energy source utilized by *M. tuberculosis* [4]–[7]. In addition, the sequencing of *M. tuberculosis* genome revealed that fatty acid β-oxidation genes are extensively duplicated [8] and are up-regulated during infection in macrophages [9] and in mice [10]. Beside fatty acids, host cholesterol is another possible carbon source used by *M. tuberculosis* during infection [11], [12].

However, *M. tuberculosis* displays a unique versatility for carbon metabolism that we are only starting to appreciate and understand. A recent study has shown that *M. tuberculosis* is not subjected to catabolic repression and is therefore capable of co-catabolizing several carbon sources simultaneously for optimal growth [13]. In particular, the simultaneous catabolism of glucose and lipids was found to potentiate bacterial growth, at least *in vitro* [13]. Therefore, even though β-oxidation is required for virulence, it is conceivable that other carbon and energy sources may be utilized by *M. tuberculosis* for optimal infection and persistence in the various microenvironments within its host. Glucose represents one of the most abundant sources of carbon and energy, and it is thus not surprising that the glycolytic pathway is highly conserved in almost all living organisms. Indication that glucose metabolism might be important for *M. tuberculosis* during infection arises from a study where putative carbohydrate transporters and a hexose kinase were found essential for infection in mice [10]. Studies in *Salmonella enterica serovar* Typhimurium, an intracellular pathogen, have also shown that glycolysis is required for infection [14], [15].

**Figure 1:**
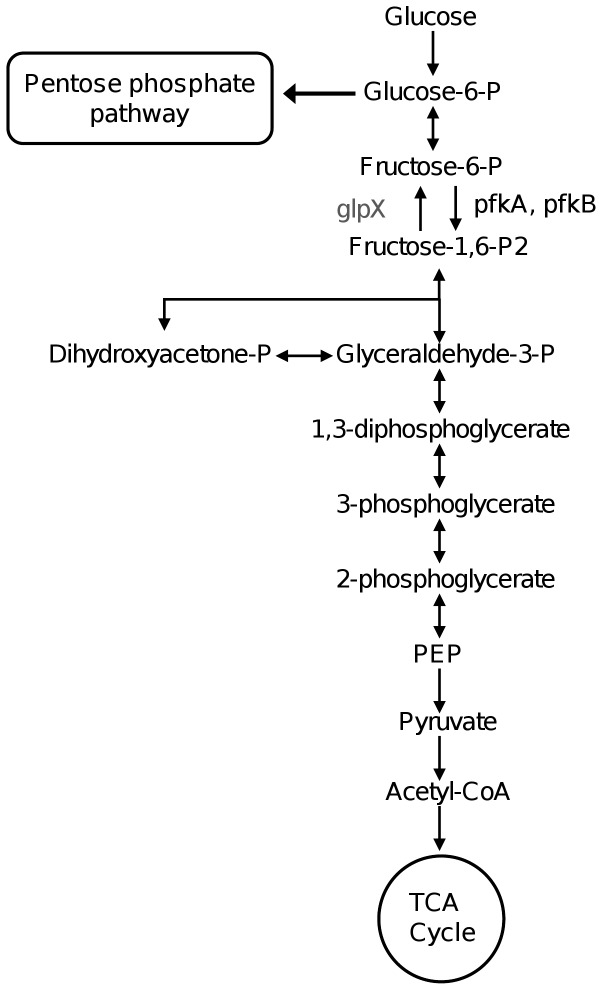
Schematic representation of the glycolytic pathway. Key committing step of glycolysis is catalyzed by *pfkA/pfkB*.

The *M. tuberculosis* genome reveals an intact glycolytic and pentose phosphate pathway but no Entner-Doudoroff pathway [8]. Early *in vitro* studies suggested that glucose is predominantly oxidized through glycolysis while a small fraction enters the pentose phosphate pathway [16]. The key-committing step of glycolysis is catalyzed by a phosphofructokinase (PFK) activity, which irreversibly catalyzes the phosphorylation of fructose-6-phosphate to fructose-1,6-bisphosphate (Fig. 1). Two putative PFKs (PFKA and PFKB) encoding genes have been annotated in *M. tuberculosis* genome, namely *Rv3010c* and *Rv2029c*, respectively. Although both enzymes are proposed to catalyze the same enzymatic reaction, they belong to different subfamilies; PFKA belongs to the PFK protein family whereas PFKB belongs to the *pfkB* subfamily of ribokinase superfamily. Furthermore, not only their amino acid sequence greatly differs, but their gene expressions are different whereby *pfkB*, is a member of the DOS regulon and is up-regulated during hypoxia [17], [18] and in activated macrophages [9]. TraSH-based mutagenesis screen indicated that both *pfkA* and *pfkB* are not essential for the survival of *M. tuberculosis in vitro* and *in vivo* growth [10], [19].

In this work, we investigated the role of PFKA and PFKB in *M. tuberculosis* glucose metabolism. We demonstrated that *pfkA* encodes a functional PFK that is essential for growth on glucose as sole carbon source, and is responsible for the total PFK activity in *M. tuberculosis*. No functional redundancy between *pfkA* and *pfkB* could be established. Our data indicate that a functional glycolytic pathway is required to limit the intracellular accumulation of glucose-derived toxic metabolic intermediates during co-metabolism. We also report a strong detrimental effect of glucose metabolism for the long-term survival of hypoxic non-replicating mycobacteria.

## Materials and Methods

### Ethics statement

All the animal experiments were approved by and carried out under the guidelines of the Institutional Animal Care and Use Committee (IACUC) of Novartis Institute for Tropical Diseases, Singapore. Non-terminal procedures were performed under anesthesia, and all efforts were made to minimize suffering.

### Mycobacterial strains and growth conditions

*M. tuberculosis* H37Rv and derivative mutant strains were grown in Middlebrook 7H9 liquid medium supplemented with 0.2% glycerol, 0.5% bovine serum albumin fraction V, 0.2% glucose, 0.085% sodium chloride and 0.05% Tween-80 or on Middlebrook 7H11 agar supplemented with 10% oleic acid-dextrose-albumin-catalase enrichment and 0.5% glycerol. When required, culture media were supplemented with hygromycin at final concentration of 80 µg/ml or with kanamycin at a final concentration of 25 µg/ml.

For growth kinetics studies in defined culture broth media, mycobacteria were first cultured in 7H9 medium until mid-log phase, washed once with defined culture broth medium (no carbon source) and then inoculated at an initial optical density at 600 nm (OD_600_) of 0.05. The defined culture broth media contained 2.5 g Na_2_HPO_4_, 1 g KH_2_PO_4_, 0.424 g glutamic acid, 1 mg pyridoxine, 0.5 mg biotin, 15 mg ferric ammonium citrate, 40 g MgSO_4_, 0.5 mg CaCl_2_, 0.6 mg ZnSO_4_, 0.6 mg CuSO_4_, 0.8 g NaCl, 0.5 g Tyloxapol and 0.1% fatty-acid free bovine serum albumin (Sigma A8806) per litre of medium. Glucose, acetate or glycerol was added at a final concentration of 0.2% as carbon source. Bacterial growth was monitored by measuring the optical density at 600 nm over time.

For growth kinetics studies in Dubos medium, mycobacteria were first culture in Dubos liquid medium (Difco) supplemented with 0.5% BSA fraction V, 0.085% NaCl and 0.03% Tween-80 until mid-log phase. The cells were inoculated at an initial OD_600_ of 0.05 in either Dubos liquid medium described previously or in complete Dubos liquid medium (further supplemented with 0.75% glucose). Bacterial growth was monitored at OD_600_ over time.

### Construction of *M. tuberculosis* knockout and complemented strains

The knockout mutants were obtained by double homologous recombination using plasmid pYUB854 as described before [20]. Briefly, fragments of ∼1 kb flanking *pfkA* or *pfkB* opening reading frames (ORFs) were PCR amplified from H37Rv genomic DNA using primer pairs: PFKA5F-PFKA5R, PFKA3F-PFKA3R, PFKB5F-PFKB5R and PFKB3F-PFKB3R (Table 1). The 5′ and 3′ flanking fragments were then cloned into pYUB854 flanking the hygromycin-resistance gene. The *sacB-lacZ* cassette excised from pGOAL17 [21] was finally cloned into the unique *Pac*I site of pYUB854. The final plasmids were UV-irradiated prior to electroporation into H37Rv strain. Positive clones of knockout mutants were selected as white colonies on 7H11 agar supplemented with hygromycin and X-Gal. Deletion of the target gene was verified by PCR (primers sequence in Table 1) and confirmed by Southern blot. Unmarked knockout mutant of *pfkA* was obtained upon removal of the hygromycin cassette using the resolvase gene-containing plasmid pWM19 as described previously [22]. Colonies were first selected on 7H11 agar containing gentamycin at 31°C and followed by selection on 7H11 agar supplemented with 2% sucrose at 39°C.

**Table 1:** Primers used in this study.

Primer	Sequence (5′-3′)	Purpose
PFKA5F	CGACTAGTCGCGCTGACCGCGACCGTCG	*pfkA* knockout
PFKA5R	TACCATGGGTACGCACCACCGCACGGATG	*pfkA* knockout
PFKA3F	GTTCTAGAAGATGGTGACGTTGCGCGGC	*pfkA* knockout
PFKA3R	CACTTAAGGTGTAACCGGCCTCGTGAAAG	*pfkA* knockout
PFKB5F	CGACTAGTCACGCAACCAGCGCTACGA	*pfkB* knockout
PFKB5R	TGCCATGGCAGTGATGTCGAGCAACCG	*pfkB* knockout
PFKB3F	GCTCTAGACGCGACGATGTGGAGAGGT	*pfkB* knockout
PFKB3R	CGCTTAAGCGCAACCGAAGCTGCGACA	*pfkB* knockout
PFKAcF	TATCTAGACCGCTACTGAGCGCCATTTA	*pfkA* ORF
PFKAcR	TAAAGCTTACCCGACGTCAACCGAAGAA	*pfkA* ORF
PFKBcF	TAAGATCTATGACGGAGCCAGCGGCGTG	*pfkB* ORF
PFKBcR	GCAAGCTTGTGTGATTGGTTCATGGCGA	*pfkB* ORF
PFKA-pET29F	TACATATGCGGATTGGAGTTCTTACCG	Cloning into pET29a
PFKA-pET29R	TACTCGAGACCGAAGAAGGCGGCGGC	Cloning into pET29a
PFKB-pET15F	TACATATGACGGAGCCAGCGGCGTG	Cloning into pET15b
PFKB-pET15R	TACTCGAGGTGTGATTGGTTCATGGCGAGG	Cloning into pET15b
PFKA-pQE60F	TACCATGGGTATGCGGATTGGAGTTCTTAC	Cloning into pQE60
PFKA-pQE60R	TAAGATCTACCGAAGAAGGCGGCGGC	Cloning into pQE60
PFKAInF	GGTCGGATTTCAGAACGGCTT	Verification of *pfkA* deletion
PFKAInR	CATGCCTACCCATCACCTCCA	Verification of *pfkA* deletion
PFKBInF	GAGCAATGCCTCGACGAACTG	Verification of *pfkB* deletion
PFKBInR	CTGCCGCGTTTCCCAAGCGA	Verification of *pfkB* deletion
PFKAusF	CGGCGTAAACCCACCTACG	Verification of *pfkA* deletion
PFKAdsR	GCGCGACAGGCTCCAAATCC	Verification of *pfkA* deletion
PFKBusF	CGCAACACCGTGGTCCGAGA	Verification of *pfkB* deletion
PFKBdsR	CTTCGACGATCTGTTCAATCC	Verification of *pfkB* deletion
HygF	CTTCACCGATCCGGAGGAACT	Verification of gene deletion
HygR	GACGACCTGCAGGCATGCAA	Verification of gene deletion
PFKAsR	ATTGCTCGACACCTCCGAGGG	Southern blot probe
PFKBsR	TTCCACGAGGTAACGCGTCC	Southern blot probe

Restriction sites are underlined.

For complementation of Δ*pfkA* mutant, the *pfkA* ORF and its putative promoter region were PCR amplified with primers PFKAcF and PFKAcR (Table 1) from H37Rv genomic DNA and cloned into the promoterless integrative vector pMV306 [23]. For complementation of Δ*pfkB* mutant, the *pfkB* ORF was PCR amplified with primers PFKBcF and PFKBcR (Table 1) and cloned into replicative vector pMV262 under the constitutive *hsp60* promoter [23]. The *Xba*I-*Hind*III fragment from recombinant pMV262- *hsp60pfkB* plasmid was then cloned into pMV306, giving pMV306-*hsp60pfkB*. All the complemented strains were verified by PCR.

### Southern blot analysis of knockout mutants

**Figure 2:**
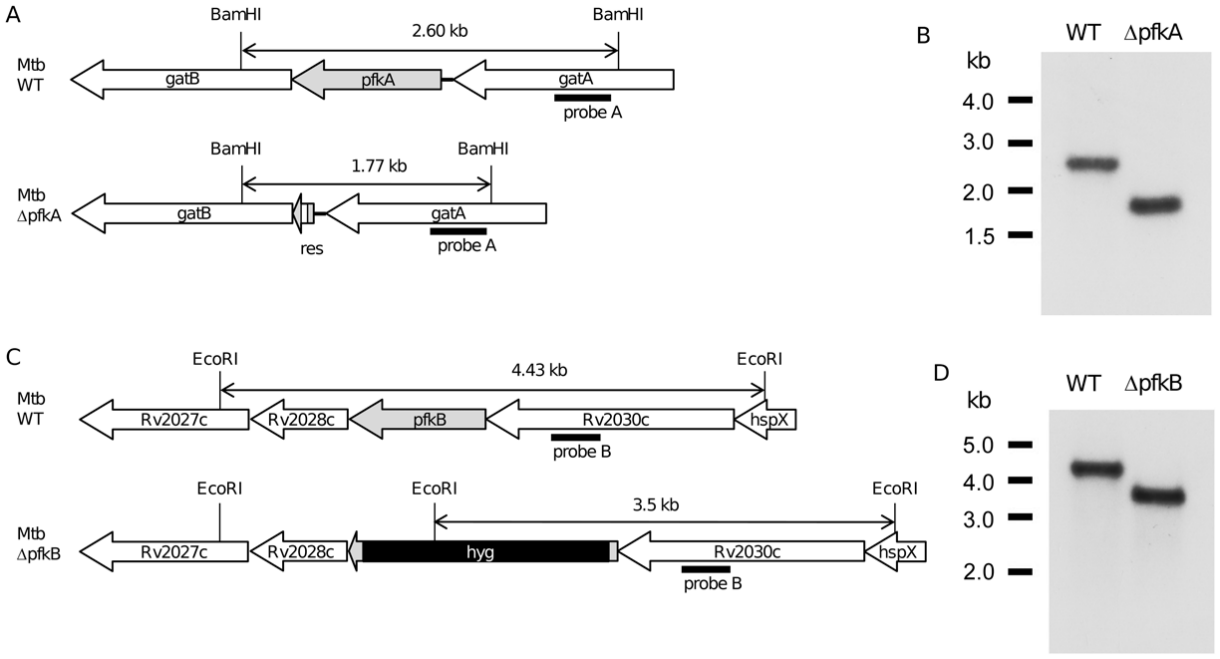
Deletion of *pfkA* and *pfkB* genes in *M. tuberculosis*. Schematic representation of the genomic regions of (A) *pfkA* in WT and ***Δ*** *pfkA* mutant, (C) *pfkB* in WT and *ΔpfkB* mutant, and location of restriction sites and probes. (B) and (D) are Southern blots confirming the knockout of *pfkA* and *pfkB* genes respectively. *res*: sites of resolvase; *hyg*: hygromycin resistance cassette.

Genomic DNA of the parental and mutant strains were digested with restriction enzyme *BamH*I for confirmation of *pfkA* deletion and *EcoR*I for confirmation of *pfkB* deletion. Digested DNA was separated on a 0.8% agarose gel, transferred onto nylon membrane and probed for modification of the loci. Southern blot analysis was performed using DIG Nonradioactive Nucleic Acid Labelling and Detection System (Roche), following the manufacturer's instruction. DIG-labelled probeA and probeB (Fig. 2) were PCR-amplified using primer pairs of PFKAusF-PFKAsR and PFKBusF-PFKBsR (Table 1) respectively. Bands were visualized using chemiluminescent detection.

### Complementation of an *E.coli* PFK knockout mutant

Mycobacterial *pfkA* and *pfkB* ORFs were PCR amplified using primer pairs PFKApQE60F-PFKApQE60R and PFKBcF-PFKBcR respectively (Table 1). *pfkA* was cloned into expression vector pQE-60 and and *pfkB* was cloned into pQE-30 (Qiagen). These vectors allow an IPTG-inducible T5 promoter-driven expression in *E. coli*. The recombinant plasmids were electroporated into Δ*pfkAΔpfkB E. coli* mutant RL257 (CGSC, Yale) [24]. The transformed RL257 strains and control RL257 strain were grown in Luria Bertani (Miller) broth medium (Difco) until mid-log phase and washed once with M9 solution (Difco). OD_600_ was then adjusted to 0.01 before streaking onto minimal medium agar containing M9 minimal salts (Difco) supplemented with 2 mM MgSO_4_ and 0.2% glucose or glycerol as carbon source. Expression of mycobacterial *pfkA* and *pfkB* genes was induced by 0.01 mM IPTG in the agar plates. Bacteria were incubated at 37°C overnight.

### Cloning, expression and purification of PFKA and PFKB

The *pfkA* and *pfkB* ORFs were PCR amplified from H37Rv genomic DNA with primer pairs PFKApET29F-PFKApET29R and PFKBpET15F-PFKBpET15R respectively (Table 1). *pfkA* was cloned into expression vectors pET-29a(+) and *pfkB* was cloned into pET15b (Novagen). *pfkA* was expressed as C-terminal 6xHis-tag recombinant protein while *pfkB* was expressed as N-terminal 6xHis-tag recombinant protein in *E. coli* BL21(DE3) (Stratagene). Bacteria cultures were grown at 37°C in LB broth until mid-log phase and then transferred to 16°C. Induction of recombinant protein expression started with the addition of 0.1 mM IPTG and bacteria were cultured at 16°C for 20 hrs. Bacterial cells were harvested and disrupted by sonication. Cell debris were removed by centrifugation and the His-tagged proteins were purified under native conditions on Ni-NTA agarose column (Qiagen) followed by size exclusion chromatography on Superdex 200 10/300 GL column (GE Healthcare). The proteins were stored at −80°C in buffer containing 50 mM Tris-HCl pH7.5 and 5 mM MgCl_2_.

### Preparation of cell-free crude extracts for enzyme and metabolite assays

Cell-free crude extracts of *M. tuberculosis* strains were prepared by harvesting mid-log phase culture grown in 7H9 medium. For metabolites measurement, bacterial strains were culture in complete Dubos liquid medium or Dubos liquid medium without glucose. Mycobacterial cells were washed twice with PBS/0.05% Tween-80 and resuspended in lysis buffer [50 mM Tris-HCl pH 7.5, 5 mM MgCl_2_, 1 mM dithiothreitol and complete protease inhibitor (Roche)]. Mycobacterial cells were disrupted mechanically by 0.1 mm glass beads in FastPrep FP220A bead-beater (Qbiogen). Lysates were clarified by centrifugation and then filtered through 0.22 µm filter. Total protein concentration was measured with BCA protein assay reagent kit (Pierce). Cell-free crude extracts to be used for metabolite assays were boiled for 10 min and centrifuged at 13,000 rpm for 10 min at 4°C.

### Phosphofructokinase activity assay

Phosphofructokinase activity was measured in an enzyme-coupled assay in which fructose-1,6-bisphophate formation is coupled to the oxidation of NADH [25]. The standard assay mixture (0.1 ml) contained 5 mM fructose-6-phosphate, 1 mM ATP, 0.3 mM NADH (Roche), 1 unit each of aldolase, triosephosphate isomerase and glycerol-3-phosphate dehydrogenase in 50 mM Tris-HCl pH7.5 and 5 mM MgCl_2_. For enzymatic assay with purified recombinant proteins, 1 mM fructose-6-phosphate and 0.1 mM ATP were used instead. The enzyme activities were measured by monitoring the decrease in absorbance at 340 nm using a SpectraMax spectophotometer (Molecular Devices) at room temperature. Quantification was done with a NADH standard curve. All enzymes and reagents used in enzyme-coupled assay were purchased from Sigma-Aldrich, unless otherwise stated.

### Immunoblotting

Samples of cell-free crude extract were separated on a NuPAGE 4–12% polyacrylamide get (Invitrogen) and transferred to PVDF membrane. PFKB was detected with rabbit polyclonal anti-PFKB antibody, raised against the recombinant PFKB, and visualized with SuperSignal West Pico Chemiluminescent Substrate kit (Pierce). Ponseus-S (Sigma-Aldrich) staining of the membrane was done to check for equal loading of the cell lysates.

### Measurement of intracellular metabolites

Intracellular metabolites concentration were enzymatically determined as described by Hasan et al [26] with slight modifications. Glucose-6-phosphate concentration was determined by measuring the increase in absorbance at 340 nm in an enzymatic assay reaction (0.2 ml) containing 0.3 mM NADP^+^ (Roche), 0.1 unit of glucose-6-phosphate dehydrogenase in 50 mM Tris-HCl pH7.5 and 5 mM MgCl_2_. To determine the concentration of fructose-6-phosphate, 0.1 unit of phosphoglucose isomerase was added to the reaction mixture after glucose-6-phosphate reaction was completed and the change in absorbance at 340 nm was recorded. Quantification was done with a NADPH standard curve. All enzymes and reagents used in metabolites measurement were purchased from Sigma-Aldrich, unless otherwise stated.

### Mouse infection

Animals were housed in specific pathogen-free conditions in individual ventilated cages in an ABSL3 facility. Female BALB/c mice of 6–8 weeks old were nasally infected with 10^3^ CFU of H37Rv parental and Δ*pfkA* strains. Four animals per group were sacrificed at the indicated time points. The lungs and spleen were aseptically harvested and homogenized in PBS/0.05% Triton X-100. The bacterial load were quantified by plating serial dilutions of the organ homogenates on 7H11 agar supplemented with cycloheximide and ampicillin each at 10 µM. The number of CFU was recorded after 16 days incubation at 37°C.

### Wayne model of hypoxia

*M. tuberculosis* H37Rv and mutant strains were subjected to slow withdrawal of oxygen as described before [27]. Mycobacteria were first cultured in Dubos liquid medium (without glucose). The cells were then diluted in either Dubos liquid medium (without glucose) or complete Dubos liquid medium to a final OD_600_ of 0.002. 17 ml of the diluted culture was aliquoted into screw-cap test tubes (20 mm by 125 mm) to maintain a head-to-space ration of 0.5 and the test tubes were tightly capped. The cultures were then stirred gently at 170 rpm on magnetic stirring platform at 37°C. Methlylene blue (1.5 µg/ml) was added to two representative tubes to monitor oxygen depletion. Growth and survival of mycobacteria were determined by enumeration of CFU after 2 to 3 weeks of incubation at 37°C on 7H11 agar plated out at various time-points..

## Results

### PfkA is responsible for the total PFK activity in *M. tuberculosis*

Two genes *pfkA* (*Rv3010c*) and *pfkB* (*Rv2029c*) have been annotated to encode a PFK in *M. tuberculosis*. To investigate the relative contribution of each gene product to the overall *M. tuberculosis* PFK activity, *M. tuberculosis* mutants deleted for either *pfkA* or *pfkB* were constructed by homologous recombination in *M. tuberculosis* H37Rv. Since *pfkA* is part of an operon, an unmarked *pfkA* KO mutant was constructed to avoid any polar effect on downstream open reading frame (ORF) *gatB* (Fig. 2). Deletion at the correct genetic locus was confirmed by Southern blot (Fig. 2). Complementation was then performed whereby an intact copy of the *pfkA* ORF and its promoter region was re-introduced into the *ΔpfkA* bacterial chromosome using the promoterless integrative plasmid pMV306. A PFK enzymatic assay was developed using cell-free extracts from the parental (WT), KO and complemented strains. Results showed that PFK activity could not be detected over background level in the Δ*pfkA* mutant (Table 2). The PFK activity in Δ*pfkB* mutant was comparable to that measured in the WT strain, suggesting that *pfkB* does not encode for a functional PFK. The PFK activity could be restored to parental level in the Δ*pfkA* mutant upon complementation with a wild-type copy of *pfkA* (Table 2). These data thus suggested that *pfkA* is responsible for the total PFK activity in *M. tuberculosis*, at least under aerobic conditions.

**Table 2:** *pfkA* encodes a functional phosphofructokinase.

Strain (H37Rv background)	PFK activity (nmol min^−1^ crude protein mg^−1^)
WT	7.2/7.4
Δ*pfkA*	nd/nd
Δ*pfkB*	7.5/7.6
Δ*pfkA* complemented with *pfkA*	7.3/7.4
Δ*pfkA* complemented with *pfkB*	nd/nd
∧His-PFKA	25.0±2.4 (nmol min^−1^ purified protein mg^−1^)
∧His-PFKB	1.7±0.02 (nmol min^−1^ purified protein mg^−1^)

Fructose-6-phosphate kinase activity of cell-free extracts from *M. tuberculosis* strains was measured by coupling fructose-1,6-bisphosphate formation to oxidation of NADH with aldose, triosephosphate isomerase and α-glycerophosphate dehydrogenase. Each biological sample was measured in duplicate. The data represent the values obtained for each duplicate of each biological sample. ∧ Enzymatic assay of purified recombinant His-tagged PFKA and His-tagged PFKB of *M. tuberculosis* was performed in triplicates and results are expressed as mean ± SD. Each experiment was repeated as least once independently and comparable values and trends were observed. Legend: nd, not detectable.

**Figure 3:**
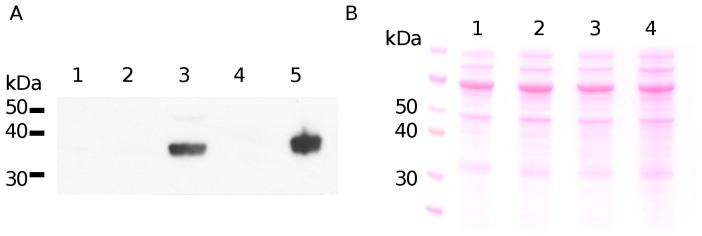
Western blot analysis of PFKB expression in wild-type *M. tuberculosis* and mutants. (A) Detection of PFKB with rabbit-anti-PFKB antibodies. (B) Ponseus-S stained of the membrane showing equal loading of cell-free extracts. Lane 1: WT; 2: *ΔpfkB* ; 3: *pfkB*-complemented *ΔpfkA;* 4: *ΔpfkA ;* 5: purified His-PFKB as control.

**Figure 4:**
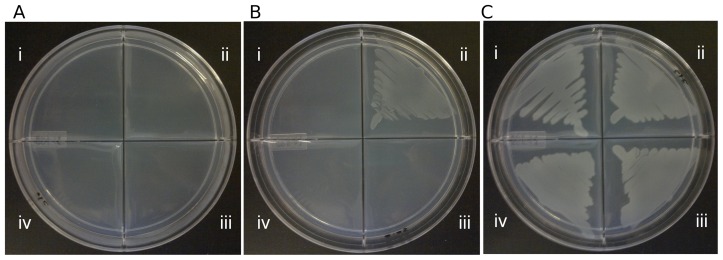
Phenotypic complementation of *ΔpfkAΔpfkB E. coli* mutant with mycobacterial *pfkA* and *pfkB*. (i) ***Δ*** *pfkAΔpfkB E. coli* RL257, (ii) RL257 complemented with Mtb *pfkA*, (iii) RL257 complemented with Mtb *pfkB* and (iv) RL257 transformed with empty vector were grown on M9 minimal agar supplemented with (A) 0.2% glucose, (B) 0.2% glucose and IPTG and (C) glycerol.

Since *pfkB* was previously reported to be upregulated under hypoxic condition and in activated macrophages [9], we hypothesized that PFKB may contribute to *M. tuberculosis* PFK activity under hypoxia but not during aerobic growth. To test whether *pfkB* encodes for a functional PFK, the *pfkB* ORF was cloned in a replicative plasmid (pMV262) under the control of the constitutive *hsp60* promoter, and expressed in the Δ*pfkA* mutant. PFKB over-expression in the Δ*pfkA* mutant was confirmed by Western blot (Fig. 3), but did not lead to detectable PFK activity levels in the cell free extracts, further supporting that PFKB does not contribute to the mycobacterial PFK activity (Table 2). Consistently, when *M. tuberculosis* PFKA and PFKB were expressed in a *pfkA/pfkB* double KO strain of *E. coli* (RL257) [24], only mycobacterial *pfkA* but not *pfkB* allowed the growth of *E. coli* RL257 on minimal medium with glucose as the sole carbon source (Fig. 4), strongly suggesting that *pfkB* does not encode for a PFK enzyme.

Finally, His-tagged PFKA and PFKB proteins were over-expressed in *E. coli*, purified and tested in enzyme-coupled assay for their PFK activity. PFKA was able to catalyze the phosphorylation of fructose-6-phosphate to fructose-1,6-bisphosphate efficiently, whereas no significant activity was detected from PFKB (Table 2).

Taken together, these data strongly support that *pfkA* is responsible for the overall PFK activity in *M. tuberculosis* H37Rv, and that *pfkB* does not catalyze fructose-6-phosphate *in vivo*.

### *pfkA* is necessary and sufficient for *M. tuberculosis* growth on glucose as sole carbon source

**Figure 5:**
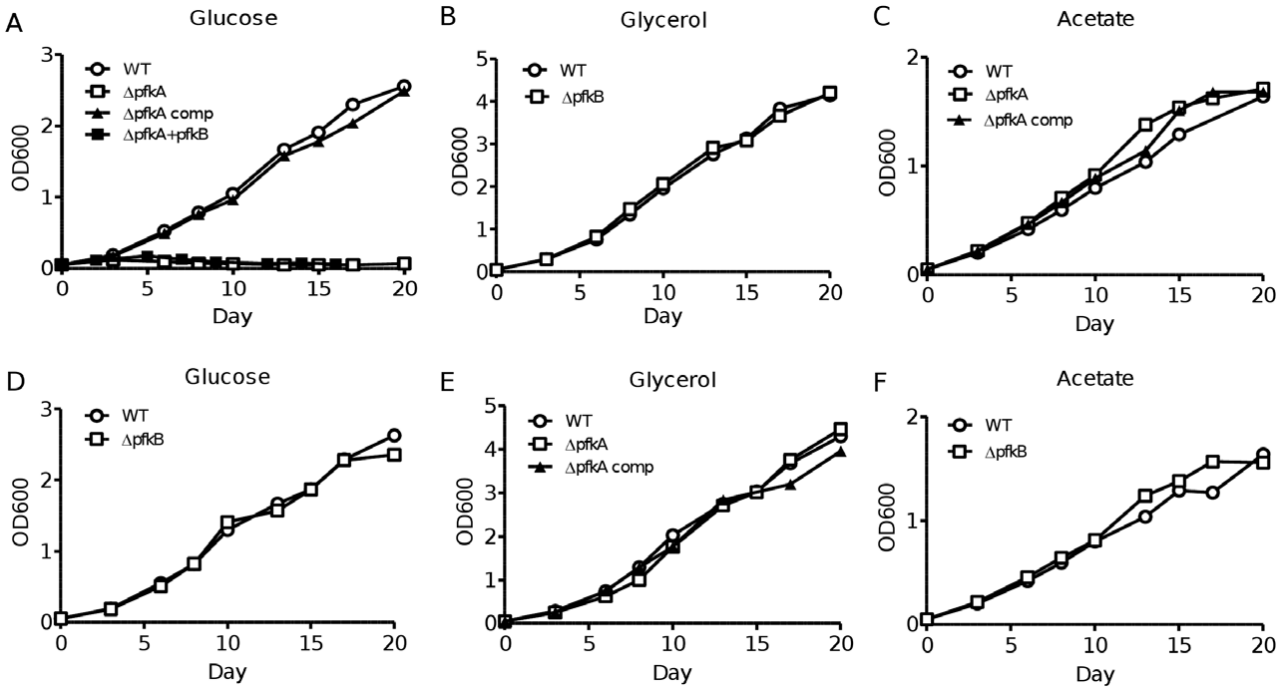
*In vitro* growth kinetic of *ΔpfkA* and *ΔpfkB* mutants on various carbon sources. Growth in liquid medium with glucose, glycerol or acetate as sole carbon source (as indicated) was monitored for Wild-type, Δ*pfkA*, Δ*pfkA* complemented with *pfkA*, Δ*pfkA* complemented with *pfkB, and* ***Δ*** *pfkB M. tuberculosis* strains (as indicated). Bacterial growth was monitored by OD absorbance at 600 nm over time. Results are representative of at least two independent experiments.

To further study the role of *pfkA* and *pfkB* in mycobacterial glucose metabolism, we tested the ability of both the Δ*pfkA* and *ΔpfkB* mutants to grow in the presence of glucose as sole carbon source. The results showed that Δ*pfkA* mutant was unable to grow efficiently on glucose as sole carbon source whereas it displayed a parental growth kinetic on acetate or glycerol (Fig. 5A–C). The growth defect on glucose observed with *μpfkA* mutant was restored upon complementation with a wild-type copy of *pfkA* (Fig. 5A). Consistent with the PFK activity data (Table 2), the growth defect of *ΔpfkA* mutant could not be reversed upon constitutive expression of *pfkB* (Fig. 5A). In contrast, *ΔpfkB* mutant showed no growth defect on glucose (Fig. 5D) which is in agreement with the PFK activity measured in this mutant strain (Table 2). These data thus further support that PFKA is indispensable for the glycolytic pathway in *M. tuberculosis* during aerobic growth with no functional redundancy with PFKB.

### PFKA is not required for virulence and survival in the mouse model of tuberculosis infection

**Figure 6:**
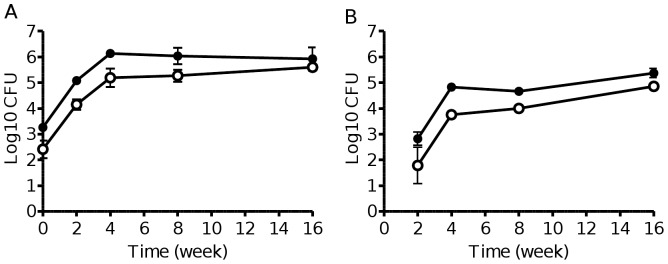
Infection profile of *ΔpfkA* mutant in mouse. 8-weeks old female BALB/c mice were nasally infected with the wild-type (black circle) or Δ*pfkA* (open circle) strains. Four animals per time point per group were used. Bacterial loads in lung (A) and spleen (B) were determined by CFU counts. Data are expressed in Log_10_ CFU per organ as the mean ± SD of four mice per group.

Genes encoding putative disaccharide transporters were predicted to be required for the first week of mouse infection [10]. Studies in *Salmonella enterica* serovar Typhimurium have shown that PFK is important during mouse infection [14], [15]. To study the role of PFKA in *M. tuberculosis* virulence, BALB/c mice were nasally infected with the parental or Δ*pfkA* strains, and their infection profiles in the lung and spleen were monitored. The results indicated that the bacterial loads in both organs recovered from both mouse groups were comparable (Fig. 6). This result thus suggested that PFKA, and therefore glycolysis, is not crucial for *M. tuberculosis* survival and persistence in the mouse lungs and spleen. This result does not rule out the possibility that PFKA may be required for survival in other animal models where pathology and physiology of the bacterium are closer to those observed during human infection.

### PFKA is required for survival of hypoxic non-replicating *M. tuberculosis*

**Figure 7:**
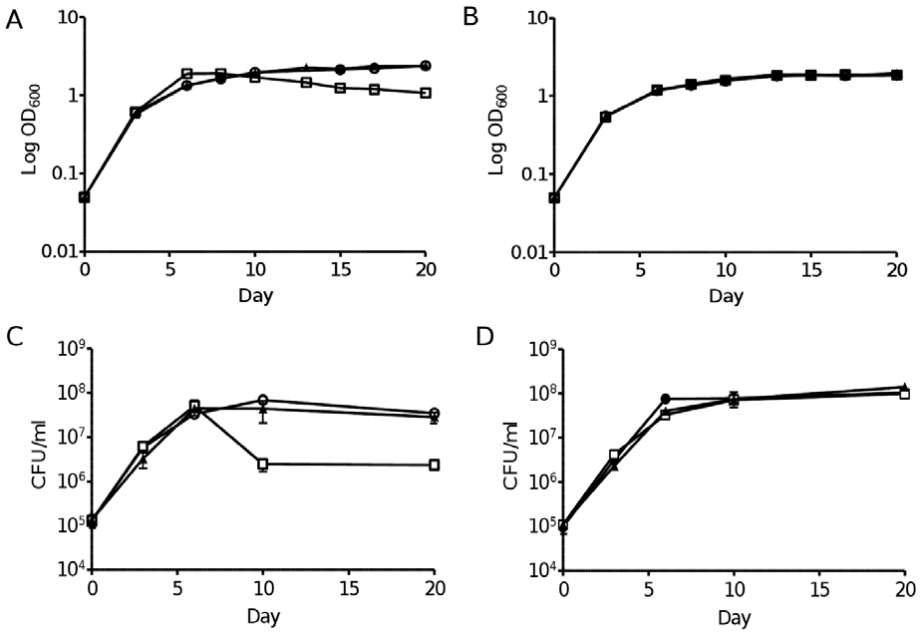
Growth kinetic of *ΔpfkA* mutant under aerobic or hypoxic conditions. Growth in aerobic (A, B) or hypoxic (Wayne model) (C, D) conditions was monitored over time for wild-type (open circle), Δ*pfkA* (open square) and complemented Δ*pfkA* (black triangle) strains as determined by OD_600 nm_ (A, B) or CFU counts (mean ± SD of triplicates) (C, D) in Dubos medium with (A, C) or without (B, D) glucose. Results are representative of at least two independent experiments.

During the course of *in vitro* aerobic growth we noticed that Δ*pfkA* mutant displayed impaired fitness upon reaching stationary phase in Dubos medium with no visible signs of clumping (Fig. 7A). Dubos medium contains large amount of glucose, amino acids and lipids as main sources of carbon and energy. When glucose was depleted from the Dubos medium, Δ*pfkA* mutant survived as well as the parental strain during the stationary phase (Fig. 7B). We thus hypothesized that accumulation of toxic glucose-derived sugar-phosphates such as glucose-6-phospate and fructose-6-phosphate in the *ΔpfkA* mutant may account for the growth defect observed during co-metabolism when oxygen tension becomes limiting. Sugar-phosphates have indeed been shown to be highly toxic in many bacteria, including *M. tuberculosis* [28]. Consistently, the pool of glucose-6-phospate and fructose-6-phosphate measured during the exponential growth of the *ΔpfkA* mutant was 50% higher compared to the parental strain (Table 3). It is interesting to note that while accumulation of sugar-phosphates occurs during the growth exponential phase, the toxic phenotype instead was only observed during the stationary phase, linking the detrimental effect of sugar-phosphate accumulation with oxygen depletion.

**Table 3:** Concentration of intracellular glucose-6-phosphate and fructose-6-phosphate in aerobic *M. tuberculosis* strains.

	Glucose-6-phosphate (µmol crude protein g^−1^)		Fructose-6-phosphate (µmol crude protein g^−1^)	
Glucose	+	−	+	−
**WT**	31.1/29.8	19.3/18.5	7.9/6.4	2.6/1.9
**Δ** ***pfkA***	55.0/55.1	32.8/31.3	14.2/13.9	3.7/3.8

Concentration of intracellular metabolites of mid-log phase *M. tuberculosis* strains cultured in complete Dubos liquid medium or Dubos liquid medium without glucose. Each biological sample was measured in duplicate. The data represent the values obtained for each duplicate of each biological sample. This experiment was repeated as least once independently and comparable values and trends were observed.

This observation prompted us to extend our study to the survival of *ΔpfkA* mutant under anaerobic conditions using the well-established *in vitro* Wayne model of hypoxia in which gradual depletion of oxygen triggers the bacterium to enter a non-replicating state [27]. In this model, Dubos medium supplemented with glucose is classically employed by the vast majority of research groups to study the physiology of hypoxic non-replicating mycobacteria [27]. The Δ*pfkA* mutant multiplied efficiently before oxygen depletion. However after day 6, which coincided with decolourization of the oxygen probe methylene blue, *ΔpfkA* bacteria displayed a significant viability loss compared to the parental and complemented strains (Fig. 7C). To test whether the attenuated phenotype was linked to the accumulation of toxic glucose-derived metabolites, the same experiment was performed in culture medium in which addition of glucose was omitted. In these culture conditions Δ*pfkA* mutant survived as well as the parental strain, demonstrating that in the presence of exogenous glucose, absence of PFK activity leads to the accumulation of toxic metabolic intermediates in hypoxic non-replicating mycobacteria (Fig. 7D).

### Glucose is detrimental for long term *M. tuberculosis* survival in the Wayne model

**Figure 8:**
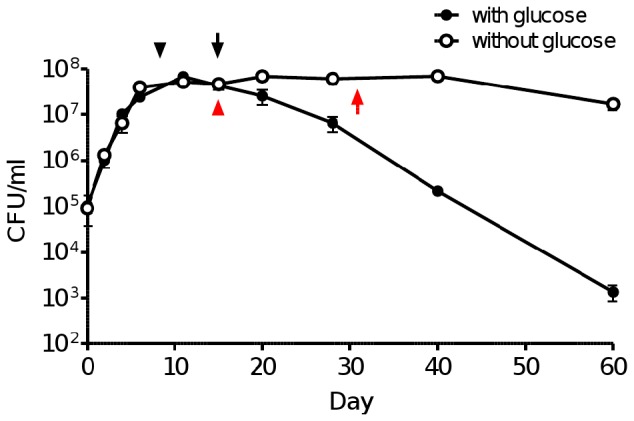
Growth kinetic of *M. tuberculosis* H37Rv under hypoxia in the presence or absence of glucose. Growth under hypoxia (Wayne model) of wild-type *M. tuberculosis* was monitored by determining the number of CFU at various time-point up to Day 60 in the presence (black square) or absence (open square) of glucose. Data are expressed as mean ± SD of triplicates. Results are representative of two independent experiments. Arrow heads mark the start of decolourization of methylene blue and full arrows mark the complete decolourization of methylene blue in culture medium with (black) or without (red) glucose.

The toxic effect of glucose observed in the Wayne model when the glycolytic pathway is disrupted prompted us to take a closer look at the limited viability traditionally observed with *M. tuberculosis* whereby non-replicating mycobacteria do not survive longer than 25 days after which they start to die at an accelerated rate with less than 0.1% of the initial inoculum of non-replicating cells still viable at day 40 [27]. While the reason for this limited long term viability has never been investigated, we hypothesized that this phenomenon might be explained by the accumulation of glucose-derived toxic metabolites over time. Consistently, when grown in medium without glucose, *M. tuberculosis* viability was maintained over 60 days, whereas a steep decrease in viability was observed in the presence of glucose (Fig. 8) as reported before. Furthermore, analysis of the intracellular metabolites pool showed significantly higher level of glucose-6-phosphate in hypoxic cells compared to non-hypoxic bacteria (Table 4). More importantly the level of glucose-6-phosphate in cells cultured in the glucose-supplemented medium was 2-fold higher than that in cells cultured in absence of glucose. Interestingly, the rate of methylene blue decolourization in culture without glucose was significantly slower than that observed in culture with glucose, suggesting that the rate of respiration in *M. tuberculosis* is slower in the absence of active glycolysis. Altogether, these observations thus indicate that long-term survival of hypoxic mycobacteria in the presence of exogenous glucose is limited by accumulation of toxic glucose-derived metabolic intermediates.

**Table 4:** Concentration of intracellular glucose-6-phosphate and fructose-6-phosphate in hypoxic non-replicating *M. tuberculosis* strains.

Glucose in medium	Glucose-6-phosphate (µmol crude protein g^−1^)		Fructose-6-phosphate (µmol crude protein g^−1^)	
	Day 6	Day 20	Day 6	Day 20
**+**	38.2/40.5	126.8/150.1	4.5/3.8	3.2/3.9
**−**	29.8/27.7	41.4/55.3	2.1/3.3	1.5/1.9

Concentration of intracellular metabolites of wild-type *M. tuberculosis* in Wayne model of hypoxia at different time points. Each biological sample was measured in duplicate. The data represent the values obtained for each duplicate of each biological sample. This experiment was repeated as least once independently and comparable values and trends were observed.

## Discussion

*M. tuberculosis* is believed to encounter a range of different microenvironments in its host as it progresses from the initial infection of alveolar macrophages to the development of granulomas and extra-pulmonary dissemination. One way that *M. tuberculosis* copes with the challenge of changing and hostile environments is by metabolic adaptation. *M. tuberculosis* is able to utilize a variety of carbon sources at least *in vitro* and genomic analysis has revealed the presence of a number of carbon metabolic pathways, including the highly conserved glycolytic pathway. Previous study has shown the presence of a functional glucose kinase which phosphorylates glucose to glucose-6-phosphate (Fig. 1) [29]. Here we demonstrated the presence in *M. tuberculosis* H37Rv of a phosphofructokinase (PFK) activity, the key regulatory enzyme of glycolysis. Similar to *E. coli*, two mycobacterial genes were annotated as PFK encoding genes, namely *pfkA* and *pfkB*. In *E. coli* PFKB is a minor isoenzyme and accounts for about 10% of the bacterial PFK activity [30]. Furthermore, a *pfkA-*deleted *E. coli* mutant was shown to be able to grow on glucose provided that PFKB was present and functional [24], [31]. Here, we have generated strong experimental evidence supporting that PFKA accounts for the total *M. tuberculosis* PFK activity without functional redundancy with PFKB. No PFK activity was detected in crude extract from *ΔpfkA M. tuberculosis* mutant; the *ΔpfkA M. tuberculosis* mutant could not be complemented with *pfkB* expressed under a constitutive strong promoter; a PFK-deficient *E. coli* mutant could be complemented when expressing *M. tuberculosis pfkA* but not *M. tuberculosis pfkB*; purified recombinant *M. tuberculosis* PFKA displayed a PFK activity *in vitro* while PFKB showed minimal activity. Although purified recombinant PFKB catalyzes fructose-6-phosphate *in vitro*, albeit at very low efficiency, it is not able to complement the loss of PFKA *in vivo*. This suggests that fructose-6-phosphate might not be the true substrate of *M. tuberculosis* PFKB. Predictive three-dimensional protein structure generated by Phyre2 server [32] showed that *M. tuberculosis* PFKB shares 40% identity with *E. coli* PFKB (data not shown). Based on the presence of the conserved catalytic motif GXGD in its amino acid sequence, *M. tuberculosis* PFKB has been classified as a member of the ribokinase superfamily, PFKB subfamily. Analysis of *T. gondii* adenosine kinase's crystal structure suggested that enzymes from the ribokinase family are able to adapt easily to a variety of sugar-based substrates [33]. Members of the PFKB subfamily which share high degree of structural conservation have been shown to phosphorylate a variety of substrates beside fructose-6-phosphate; examples are fructose-1-phosphate in *E. coli* [34], [35] and tagatose-6-phosphate in *S. aureus* [36] and *E. coli*, although with a lower efficacy than fructose-6-phosphate [37]. Thus, it is possible that *M. tuberculosis* PFKB is able to phosphorylate sugar-based substrates other than fructose-6-phosphate. So far none of the studies on the kinases from the PFKB subfamily have identified amino acid residues involved in substrate specificity. As such the nature of the *M. tuberculosis* PFKB substrate cannot be deduced from its amino acid sequence and has yet to be elucidated.

Co-metabolism experiments showed a defect in cell viability with Δ*pfkA* mutant upon entry into the stationary phase. The attenuation phenotype was correlated with accumulation of toxic metabolic intermediates in the glycolytic pathway upstream of PFKA. Consistently, removal of glucose from the culture medium restored viability of the Δ*pfkA* mutant. Significantly higher intracellular pools of glucose-6-phosphate and fructose-6-phosphate in the Δ*pfkA* mutant, compared to the parental strain, further supports the hypothesis that these sugar-phosphates may be toxic to the bacterial cell and accumulate in a PFK-deficient mutant. This finding is consistent with a previous study where we showed that excessive metabolic intermediates such as glycerol phosphate, dihydroxyacetone phosphate and methylglyoxal are toxic to *M. tuberculosis* [28]. Accumulation of sugar-phosphate may have various physiological consequences including mRNA destabilization [38], stimulation of gene expression [39], and activation of pyruvate kinase [40], all of which may contribute to impair the cell viability. The toxic effect of sugar-phosphate in *M. tuberculosis* was previously reported whereby accumulation of maltose-1-phosphate leads to bacterial death *in vitro* and in mice [41]. It is of interest to note that maltose-1-phosphate is a product of trehalose catabolism with glucose-6-phosphate being a precursor of trehalose, thereby linking glycolysis and trehalose pathway. In addition to the accumulation of toxic metabolic intermediates, the impact of glycolysis disruption on other metabolic pathways may also play a role in reduced cell viability. Also, it must be noticed that the Δ*pfkA* mutant did not exhibit significant growth defect in standard 7H9 medium including during the stationary phase (data not shown). This discrepancy between Dubos and 7H9 media may be attributed to the higher concentration of glucose in Dubos medium compared to 7H9 (0.75% versus 0.2%), with the idea that a threshold glucose concentration may be necessary before a toxic effect can be observed. However, other differences in the composition between both media may also explain the difference in the phenotype observed.

Interestingly, a detrimental effect of glucose was observed in wild-type *M. tuberculosis* grown *in vitro* under hypoxia in the well-established Wayne model. We showed that *M. tuberculosis* could persist in a non-replicating state for much longer when glucose was omitted in the culture medium. There has been much speculation on the possible reasons of the limited persistence of *M. tuberculosis* in the NRP2 phase of the Wayne model, such as nutrients exhaustion or low levels of ATP. Here we show that the limited mycobacterial persistence is linked to the presence of glucose in the medium and is likely due to the accumulation of glucose-derived toxic metabolic intermediates. We believe that this finding is of great importance and advocates for revisiting the mechanisms employed by *M. tuberculosis* for long-term persistence in the absence of growth.

The metabolomic profile of *M. tuberculosis* infected murine tissues was recently analyzed and revealed that the level of glucose and glycogen in those tissues decreased along with the increase in phospholipids level [42]. This may suggest that *M. tuberculosis* switches from the carbohydrate to lipid metabolism in order to adapt to its microenvironment. However, we did not observe any significant difference between the Δ*pfkA* and parental strains in their ability to colonize and persist in the mouse lungs and spleen. This suggests that the glycolytic pathway is dispensable during mice infection and also indicates that the toxic effect observed *in vitro* in Dubos medium is not observed *in vivo*. It is therefore possible that *M. tuberculosis* replicates and persists in an environment where access to glucose is limited. Alternatively, since attenuation of the Δ*pfkA* mutant was seen mostly under hypoxia *in vitro*, absence of hypoxic granuloma or lesions in mice may not allow recapitulating such attenuation [43]. It would be interesting to determine the fitness of *ΔpfkA* mutant in animal models where hypoxic granulomatous lesions are formed. Regardless, the maintenance of an intact glycolytic pathway in *M. tuberculosis* throughout evolution indicates that glycolysis could play an important role in mycobacterial infection and persistence in microenvironments not recapitulated in mice.

In conclusion, we provide here the experimental evidence that PFKA is responsible for the overall PFK activity in *M. tuberculosis* and that there is no functional redundancy with PFKB. Furthermore, our work demonstrates that in the presence of exogenous glucose, hypoxic mycobacteria tend to accumulate toxic glucose-derived metabolic intermediates that impair the bacilli long-term survival. Disruption of the glycolytic pathway further accentuates accumulation of these toxic intermediates.
