# Correction: Characterization of Phosphofructokinase Activity in *Mycobacterium tuberculosis* Reveals That a Functional Glycolytic Carbon Flow Is Necessary to Limit the Accumulation of Toxic Metabolic Intermediates under Hypoxia

**DOI:** 10.1371/annotation/7197763f-327f-4d6c-89fd-fba317e52c18

**Published:** 2013-11-04

**Authors:** Wai Yee Phong, Wenwei Lin, Srinivasa P. S. Rao, Thomas Dick, Sylvie Alonso, Kevin Pethe

The last author, Kevin Pethe, should be noted as a Corresponding Author for the article. The last author's email address is: kevin.pethe@ip-korea.org

